# Performance Characterization of Watson Ahumada Motion Detector Using Random Dot Rotary Motion Stimuli

**DOI:** 10.1371/journal.pone.0004536

**Published:** 2009-02-19

**Authors:** Siddharth Jain

**Affiliations:** EECS Department, University of California, Berkeley, California, United States of America; University of Southern California, United States of America

## Abstract

The performance of Watson & Ahumada's model of human visual motion sensing is compared against human psychophysical performance. The stimulus consists of random dots undergoing rotary motion, displayed in a circular annulus. The model matches psychophysical observer performance with respect to most parameters. It is able to replicate some key psychophysical findings such as invariance of observer performance to dot density in the display, and decrease of observer performance with frame duration of the display.

Associated with the concept of rotary motion is the notion of a center about which rotation occurs. One might think that for accurate estimation of rotary motion in the display, this center must be accurately known. A simple vector analysis reveals that this need not be the case. Numerical simulations confirm this result, and may explain the position invariance of MST(d) cells. Position invariance is the experimental finding that rotary motion sensitive cells are insensitive to where in their receptive field rotation occurs.

When all the dots in the display are randomly drawn from a uniform distribution, illusory rotary motion is perceived. This case was investigated by Rose & Blake previously, who termed the illusory rotary motion the omega effect. Two important experimental findings are reported concerning this effect. First, although the display of random dots evokes perception of rotary motion, the direction of motion perceived does not depend on what dot pattern is shown. Second, the time interval between spontaneous flips in perceived direction is lognormally distributed (mode≈2 s). These findings suggest the omega effect fits in the category of a typical bistable illusion, and therefore the processes that give rise to this illusion may be the same processes that underlie much of other bistable phenomenon.

## Introduction

Many models of visual motion perception have been proposed [1], [2], [3], [4], [5]. Although much research has since been done on studies of human visual motion perception, little work has been done to psychophysically characterize the performance of these models. This is important for obvious reasons. A correct model of motion sensing should match human psychophysical performance on motion detection, and also agree with what is known currently about the neurophysiology of motion sensitive cells in the brain.

This paper presents a psychophysical performance characterisation of Watson & Ahumada's model of visual motion sensing [3], the first one to do so in my knowledge. The ability of Watson Ahumada motion detector to detect motion in random dot kinematograms is compared against human psychophysical performance. The stimulus, termed the racetrack, consists of random dots displayed in a circular annulus. The dot pattern is refreshed periodically, and a certain fraction of dots are correlated to move either clockwise (CW) or counter-clockwise (CCW) in the next frame. By varying the fraction of dots to be correlated, the amount of motion signal in the display can be controlled (see Movies S1, S2, S3 for illustration). There are many other parameters that can be varied, and performance of both the model and human observers is measured.

The model is able to match human performance with respect to most, but not all, stimulus parameters. For example, it is found that human observers are insensitive to the dot density in the display. The model shows similar behavior. The invariance of observer performance to dot density provides strong evidence against motion models based on matching dots to their nearest neighbors in the next frame [6], [7]. Such models predict that observer performance should decrease with increase in dot density, according to the probability of mismatch formula [8]. This is because as the dot density increases, the chances that the nearest neighbor is not in fact the correlated partner from the previous frame increase. Another experimental finding is that a frame duration of about 30 ms is found to be optimal for motion perception. I explain this result in terms of the spatiotemporal receptive field (STRF) structure of motion sensitive cells. At any time instant *t*, the response of such cells is roughly based on the value of the spatiotemporal stimulus from time *t-T* to *t*, with *T* of the order of 200 ms. When the frame duration is of the order of *T* or higher, the input is mostly constant within a window of *T* ms and therefore the cells fail to detect any motion. On the other hand if frame duration is very low the input may be changing at a rate that the cells cannot handle. This will again result in failure of cells to respond optimally.

The motion in racetrack is rotary as opposed to the more commonly encountered translational case. Associated with the concept of rotary motion is the notion of a center about which rotation occurs. One might think that for accurate estimation of rotary motion in the display, this center must be accurately known. A simple vector analysis presented in this paper reveals that this need not be the case. Numerical simulations confirm this result, and may explain the position invariance of MST(d) cells. Position invariance is the experimental finding that cells that are sensitive to rotary motion are insensitive to where in their receptive field rotation occurs [9].

A special case of the racetrack is when all dots are randomly drawn from a uniform distribution in each frame, i.e., there are no correlated dots. One would expect that in this case the perception would be that of random twinkling noise, since there is no motion embedded in the stimulus. However, about two-thirds of observers report perception of rotary motion. This illusory motion was investigated by Rose & Blake previously, who termed the phenomenon the omega effect [10]. The omega effect is a classic example of paternicity, the tendency of the brain to find meaningful patterns in meaningless noise [11]. Two important results concerning this effect are reported in this paper. First, although the display of random dots evokes perception of rotary motion, the direction of motion perceived does not depend on what dot pattern is shown. Second, the time interval between spontaneous flips in perceived direction is lognormally distributed (mode≈2 s).

It may be worthwhile to mention some aspects of the “Materials & Methods” in this paper that are distinct from the traditional psychophysics paradigm. In the experiments described here, each trial has a 60 s duration. During this time, the direction of rotation changes randomly and the observer is faced with the task of continuously tracking the direction of rotation. Observer performance is calculated by cross correlating observer response with actual direction of rotation. The maximum value of the normalized cross correlation function denoted by χ is taken to be a measure of observer performance. This method is distinct from traditional psychophysics paradigm, in which the display is shown to observer for fraction of a second, and the observer has to judge if motion was perceived CW or CCW. After many trials the confusion matrix and *d′* is calculated [12]. The reason for the new method is none, except that it naturally occurred to me. Also it is my opinion that sub-second trial duration may not provide enough time for visual system of observer to reach steady state. One would expect that trial duration should be such that the percent correct and *d′* should be independent of trial duration. This can only happen if the system is in steady state. A side-benefit of the new method is that it enables the calculation of reaction time of the observer. This is the time delay at which the normalized cross correlation function reaches its maximum value. It is found that for most observers, reaction time ranges from 0.5–2 s depending on how easy it is to detect motion in the display.

In summary, the paper can be said to have three main contributions:

1. It presents a psychophysical performance characterisation of Watson & Ahumada's model of visual motion sensing. The model is found to provide a good fit to the experimental data for most, but not all, stimulus parameters.
2. It shows that for accurate estimation of rotary motion in a display, it is not necessary that the center of rotation be accurately known. This may explain the fact that rotary motion sensitive cells found in MST/MSTd areas of the brain are insensitive to where in their receptive field rotation occurs.
3. It presents two experimental findings concerning the omega effect. First, observer response is irreproducible. Second, the time interval between spontaneous flips in perceived direction is lognormally distributed (mode≈2 s). These findings suggest the omega effect fits in the category of a typical bistable illusion, and therefore the processes that give rise to this illusion may be the same processes that underlie much of other bistable phenomenon.

### Previous Work

Visual motion perception has been a heavily researched topic and hence this paper will necessarily limit itself to a discussion of the most relevant work. Reviews reflecting the state-of-the-art in this area can be found in [13], [14], [15], [16]. Three seminal models of visual motion perception were proposed by Adelson and Bergen (1985), van Santen and Sperling (1985), and Watson and Ahumada (1985) [1], [2], [3]. Central to the Adelson Bergen & Watson Ahumada models is the concept that the entire power spectrum of an image undergoing coherent translation lies on a plane in the spatiotemporal frequency domain [17]. Determining this plane is therefore equivalent to determining the motion of the image. In its original form the Adelson Bergen motion detector is limited to detecting motion in 1D. Its extension to 2D was provided by Heeger (1987), Simoncelli and Heeger (1998) [18], [19]. The model has been refined further in Rust, Mante, Simoncelli, and Movshon (2006) where it is shown that it can capture the full range of pattern motion selectivity found in MT [20]. Emerson, Bergen, and Adelson (1992) did a study in which it was shown that the responses of V1 complex cells from cat's striate cortex were well fitted by the Adelson Bergen model [21]. Moreover, cell responses were found to be inconsistent with the van Santen and Sperling model. Cells sensitive to rotary motion have been discovered in areas MST/MSTd of the brain [22], [23], [9]. These cells have large receptive fields compared to cells in V1/MT. Also, they are not sensitive as to where in their receptive field rotation occurs, a phenomenon termed position invariance [9].

Random dot kinematograms (RDKs) have been widely used in studies of visual motion perception [6], [7], [8], [10], [24], [25], [26], [27], [28]. Newsome & Pare (1998) have remarked that random dot displays are useful because they stimulate primary motion sensing mechanisms while minimizing familiar positional cues [24]. Newsome, Britten, & Movshon (1989) found that a dot correlation of at least six percent is required for monkeys to be able to detect motion in RDKs undergoing translational motion [25]. The present study gives a similar result for human observers. The effect of time-sampled displays on motion perception has been previously researched by Morgan (1980), Watson, Ahumada, & Farrell (1986) [29], [30]. Williams & Sekuler (1984) had studied the effect of dot density on observer performance [8]. They formulated the probability of mismatch formula according to which observer performance should decrease with increase in dot density, a view challenged by the present paper.

A special case of the racetrack is the omega effect, in which a display of dynamic uniformly distributed random dots in a circular annulus evokes perception of illusory rotary motion. This phenomenon was discovered by Rose & Blake (1998) [10] although they trace its origin to as far back as Mackay (1965) [31]. Recently several papers studying illusory motion from Glass patterns have appeared in the literature [32], [33], [34], [35]. Motion perception in such cases, where the spatial form of the stimulus is believed to guide motion perception, has generally been termed as implied motion in order to distinguish it from real motion, in which the display itself contains non-zero motion energy. Geisler (1999) had suggested motion streaks as providing a spatial cue that guides motion perception [36]. Barlow & Olshausen (2004) have explained the phenomenon of motion streaks and flow seen in Glass patterns by pointing out that the power spectrum of a motion blurred image or a Glass pattern exhibits strong anisotropy, which is a characteristic property of a moving image, and therefore excites the mechanisms that normally detect the distortions of local power spectrum caused by motion [37]. It is to be noted that the omega display does not display the anisotropy in power spectrum associated with Glass patterns, yet rotary motion is seen in it.

## Materials and Methods

**Figure 1:**
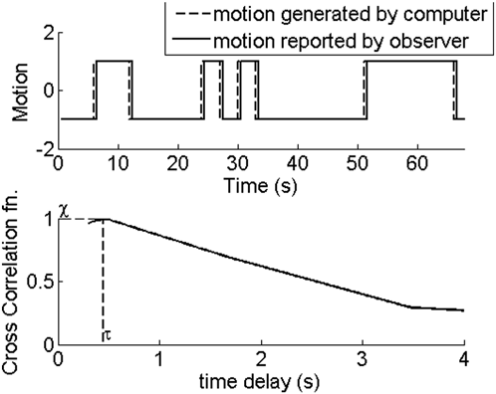
The dotted curve is the motion generated by the computer, and the solid curve is the motion reported by the observer. χ is the maximum value of the normalized cross correlation function and τ is the time delay at which χ occurs.

The experimental stimulus used in this study is termed racetrack. Three movies of the stimulus are included with this paper. A Java applet is also available online at http://purl.oclc.org/NET/racetrack. The racetrack stimulus consists of a random dot pattern displayed in a circular annulus. The dot pattern is refreshed periodically. A certain fraction *c* of the dots, referred to as correlated dots, are rotated by an angle *θ* in the next frame. The remaining dots have their positions generated randomly and uniformly in Cartesian *(x,y)*, and are representative of noise. The algorithm for generating dots is such that if a dot is correlated in the present frame, it is guaranteed not to be correlated in the next frame. This eliminates the appearance of multiple dot trajectories, and thus the only motion cues in the stimulus are two dot apparent motion cues. Observers see a swarm of dots that appears to rotate clockwise (CW) or counter-clockwise (CCW). The direction of rotation changes randomly according to the polarity of a coin that flips every 3 s. They are instructed to click the left mouse button for CCW motion, and the right mouse button for CW motion. By cross correlating the observer response with the actual direction of rotation of the correlated dots, an estimate of observer performance and reaction time denoted by χ and τ respectively is obtained. This process is illustrated in Figure 1. χ is defined as the maximum value of the normalized cross correlation function. τ is the time delay at which χ occurs. A χ value of 1 indicates perfect detection of the embedded motion. At *c = 0* the observer response can still be cross correlated with the input signal, which would have dictated the rotation of correlated dots if there were any in the stimulus. The χ value obtained in this case reflects chance, or zero, detectability of embedded motion. Response reproducibility is quantified by cross correlating observer response curves in response to the same stimulus in multiple trials.

Definitions and default values of some parameters are as follows: dot correlation *c* = number of correlated dots/total number of dots; frame duration *fd* = length of time for which a frame stays on screen, default = 30 ms; dot density *dd* = dots per unit area, default = 5 dots/degrees^2^; angle of rotation *θ* = angle by which correlated dots are rotated, default = 5°, the spatial hop size of a correlated dot = *rθ* where *r* is distance of dot from center, *θ* in radians; inner circle diameter *ic* = angle subtended by inner circle diameter at the eye, default = 7°; outer circle diameter *oc* = angle subtended by inner circle diameter at the eye, fixed at 10° in all experiments; dot diameter = 5′, fixed in all experiments; duration of a trial = 60 s. Stimuli were displayed on a NEC MultiSync FP1370 22″ (20″ viewable image size) CRT monitor with display resolution = 640×480@100 Hz; black dots (luminance≈0) against a background luminance of 10.8 cd/m^2^ were displayed; viewing distance = 1.65 m. The range and default values of some parameters is summarised in Table 1.

**Table 1:** Default values and range of various parameters used in experiments.

Parameter	Range	Default value
Dot correlation c	0–0.5	-
Frame duration fd	10–100 ms	30 ms
Dot density dd	1–25 dots/degrees^2^	5 dots/degrees^2^
Angle of rotation θ	1–20°	5°
Inner circle diameter ic	1–9.5°	7°
Outer circle diameter oc	-	10°
Dot diameter	-	5′
Duration of a trial	-	60 s
Dot luminance	-	≈0
Background luminance	-	10.8 cd/m^2^
Monitor resolution	-	640×480 pixels
Viewing distance	-	1.65 m

The study was conducted over a period of several years, and new observers were recruited as old ones dropped out. In all experiments the number of observers is at least four, and number of trials ≥20 for each data point shown in the figures. Error bars in the figures equal one standard deviation (s.d.), unless otherwise stated. Custom software was written by the author in C# to generate the stimuli. The study was approved by Committee for Protection of Human Subjects (CPHS), UC Berkeley. Written informed consent was obtained from subjects.

### Model Description

**Figure 2:**
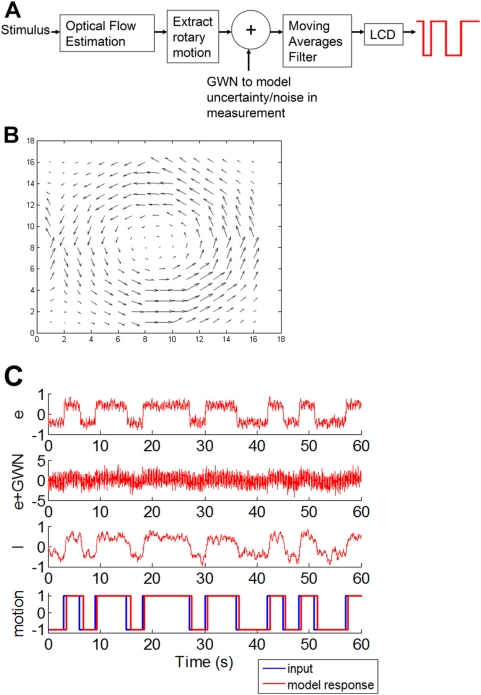
(a) Block schematic of the model (b) Optical flow output by Watson Ahumada motion detector (c) Model response at various other stages in the pipeline.

The following steps and Figure 2 describe the complete pipeline used to model observer responses to the racetrack:

- Step 1: Stimulus is input to the Watson Ahumada motion detector, which at time *t* gives the instantaneous optical flow.
- Step 2: The optical flow is easily converted into a measure of rotary motion signal by taking cross products with radial vector, followed by weighted averaging. The weights are obtained in Step 1; for each velocity estimate the Watson Ahumada detector is able to provide a confidence/error measure which is used as the weight. Area MST(d) in the brain is believed to carry out this type of processing, where the local motion signals from stage MT are pooled to estimate global patterns of rotation and expansion that guide in heading estimation [9], [38], [39]. The output of this step is denoted by *e(t)*.
- Step 3: The human visual system must integrate information over a certain interval of time to compute a reliable estimate of motion. This is achieved by passing *e(t)* through a moving averages filter, with window size of half a second. The output of this step is denoted by *I(t)*.
- Step 4: While doing psychophysical experiments with human observers, the only information available is the direction in which the observer is perceiving motion. Therefore in order to compare model response with experimental psychophysics, *I(t)* is passed through a level crossing detector (LCD) with thresholds ±B. B = 2σ(I) at *c = 0* under default parameters. This choice of B makes the events when *I(t)* may cross detection threshold, given there is no rotary motion in the stimulus, unlikely. The behavior of level crossing detector is as follows: when input crosses +/−B the detector signals CCW/CW motion respectively, and continues to do so until the input crosses threshold in the opposite direction. When that happens, the LCD flips to the opposite state.

χ, τ can now be computed for the model, and values compared to experimental psychophysics. In its present form the model is strictly deterministic. However, the human visual system necessarily exhibits some variability, characteristic of any real world physical system. In fact as shown in the results section, it is found that at *c = 0* observer responses are not reproducible. This variability in response is incorporated into the model by adding Gaussian White Noise (GWN) *n(t)* to *e(t)*, until the model response reproducibility also drops to zero at *c = 0*. This occurs for *k = σ(n(t))/σ_0_*≥approximately six, where *σ_0_* stands for *σ(e)* at *c = 0* under default parameters. Accordingly *k* was fixed at six. Model simulations were run at a resolution of 128×128 pixels, unless otherwise stated. Circular dots in psychophysical experiments were approximated as squares of equal area in model simulations. In all results, Watson Ahumada sensors are tuned to a center frequency of 0.64 cycles/degrees, unless otherwise stated. The reason for this setting is that it gave acceptable results. It may also be noted that most of the energy in power spectra of natural images is concentrated at relatively low spatial frequencies [40]. Default values of model parameters are summarised in Table 2. My source code for the Watson Ahumada component of the model is publicly available [41].

**Table 2:** Default values of model parameters used in simulations.

Parameter	Default value
Spatiotemporal filters	As in Watson & Ahumada (1985) [3]
Center frequency	0.64 cycles/°
Noise	σ(n(t))/σ_0_≈6
LCD threshold B	2σ(I) at c = 0
Moving averages filter	Impulse Response 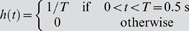
Resolution	128×128 pixels

## Results

We begin with a discussion of the omega effect (*c = 0* case of racetrack), and present two important results. First, although the display triggers perception of rotary motion, the direction of motion perceived is not dependent on what dot pattern is shown. Second, the time interval between spontaneous flips in direction exhibits a lognormal distribution.

### Omega effect: Response Reproducibility and distribution of spontaneous flips in perceived direction

As mentioned earlier, the omega effect is the *c = 0* case of the racetrack. About two-thirds of observers report perception of rotary motion at *c = 0*, even though there is no motion embedded in the stimulus [42]. The perceived direction of motion changes randomly from time to time. After prolonged viewing most observers can usually choose the perceived direction of motion at will. For some observers the direction of motion switches when a sudden attention grabbing stimulus is given (such as a sudden tap on the back of the head). Some observers have even remarked that mere pressing of a mouse button causes the perceived direction of motion to reverse.

An important characteristic of the omega effect is that an observer gives different responses to the same stimulus in multiple trials. This is quantified in the following way. The observer response curves in response to the same stimulus in two separate trials are cross correlated. Let ζ denote the maximum value of the normalized cross correlation function. ζ is taken to be the measure of response reproducibility. It is found that the value of ζ when the same stimulus is shown in multiple trials is no different than the value of ζ when different stimuli are shown in multiple trials. Thus, the response reproducibility of the omega effect is zero. This may happen because the display is inherently ambiguous like most, if not all, bistable illusions. Both interpretations are equally likely and the brain randomly chooses a configuration at any time instant. It is found that ζ = μ_1_±σ_1_ = 0.145±0.1048 (mean±s.d.) based on 47 trials in which same stimulus is shown from trial to trial. Further, ζ = μ_2_±σ_2_ = 0.118±0.1359 based on 67 trials in which different stimuli are shown in multiple trials. One sided *t*-test to test the null hypothesis μ_1_ = μ_2_ against the alternate hypothesis μ_1_>μ_2_ gives *t* = 1.196. At α = 0.05 level of significance the null hypothesis cannot be rejected (*P* value = 0.1158).

**Figure 3:**
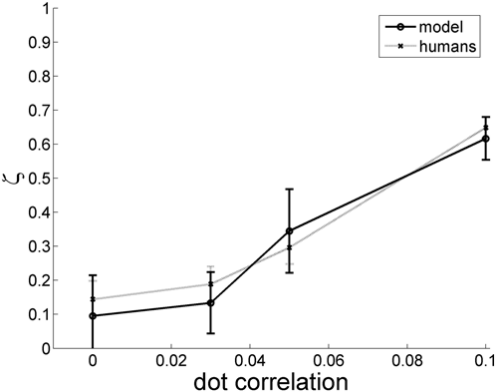
Response reproducibility ζ vs. c. Both model and humans show zero reproducibility at c = 0, and the reproducibility steadily increases with c, because the motion signal gets stronger.

The foregoing discussion has shown that the reproducibility of response is zero for the omega effect (*c = 0*). However, intuitively we expect if c is not zero, i.e., some dots are deliberately correlated to undergo rotary motion, then observers should start responding in direction of motion of correlated dots. Figure 3 shows the response reproducibility increases with c as expected (ζ = 1 reflects perfect reproducibility).

**Figure 4:**
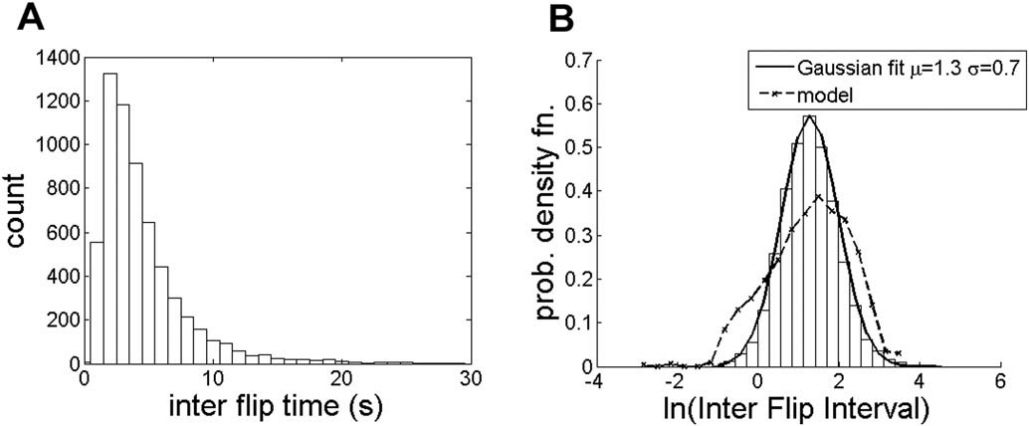
(a) histogram of Inter Flip Interval (IFI) at c = 0 (mode≈2 s) (b) normalised histogram of ln(IFI) together with a Gaussian fit. The pdf of ln(IFI) given by the model is also shown.

Figure 4(a) shows the histogram of the inter flip interval (IFI), which is the time interval between spontaneous reversals in perceived direction of motion, at c = 0. The mode of the histogram occurs at IFI≈2 s. The histogram is well approximated by a lognormal distribution which is evident in Figure 4(b), where the pdf (probability density function) of ln(IFI) is plotted together with a Gaussian fit. The IFI of many bistable illusions is lognormally distributed. Such distributions are common in biology, and one way to interpret them is in terms of the noise driven motion of a state point [43].

The mechanisms underlying omega effect are not clear. When dots are displayed in a circular annulus their freedom of movement is restricted. The dots at the boundary cannot move in all 360° directions. In the limit when the annulus width tends to zero, the dots can only move tangentially. This suggests an increase in the omega effect with decrease in annulus width which is experimentally true [10]. When the annulus has appreciable width the dots at boundary are more likely to bounce normal to the boundary. Some observers do report perception of a radial pulsating motion in the omega display [42]. Rose&Blake (1998) postulated that the omega effect arises because of interaction between cells that are sensitive to curvature in the display, and cells that are sensitive to motion [10]. For its part, the Watson Ahumada model outputs a zero mean white noise like signal in response to the omega display, since there are dots bouncing off in all directions randomly. This signal combined with the intrinsic noise *n(t)* (which at *c = 0* is six times stronger than the Watson Ahumada signal) results in rapid zero mean fluctuations. Because of their stochastic nature, these fluctuations become large enough at times to cross the LCD thresholds. The IFI distribution resulting from such stochastic fluctuations is also shown in Figure 4(b) for comparison.

### Effect of dot correlation c

**Figure 5:**
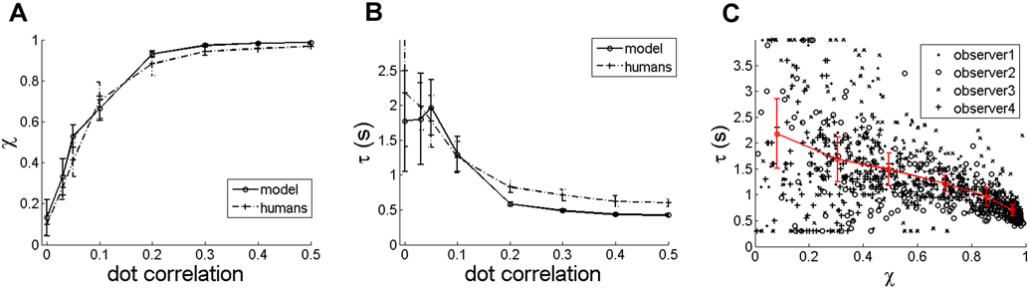
(a) χ vs. c (b) τ vs. c (fd = 30 ms, ic = 7°, dd = 5 dots/degrees^2^) (c) τ vs. χ scatter plot and piecewise linear fit for experimental data.

Figure 5(a) shows variation of signal detectability χ vs. the dot correlation *c*. A χ value of 1 means perfect detection, and χ at *c = 0* reflects the baseline zero level of χ corresponding to chance detectability. The increase in χ with c is easy to understand, as the value of c directly controls the amount of motion embedded in the stimulus. As can be seen from the figure, the model fits the experimental data very closely. If the threshold for motion perception is defined as the value of *c* for which χ is one standard deviation higher than the χ value at *c = 0*, then this gives a threshold of c in the range of 0.03 to 0.06. This is comparable to thresholds reported elsewhere [24], [25]. The experimental method described in this paper allows the measurement of the reaction time τ of an observer. Figure 5(b) shows a graph of the reaction time τ vs. *c*. For c≈0, τ is about 1.5 s, and decreases steadily with increase in *c*. It takes less time to recognize the motion signal as the signal gets stronger. At high values of *c*, τ is about half a second. The model is seen to fit the experimental data well. In general χ and τ are inversely correlated as shown in Figure 5(c). Parameters that tend to increase χ tend to decrease τ and vice-versa.

### Effect of frame duration fd

**Figure 6:**
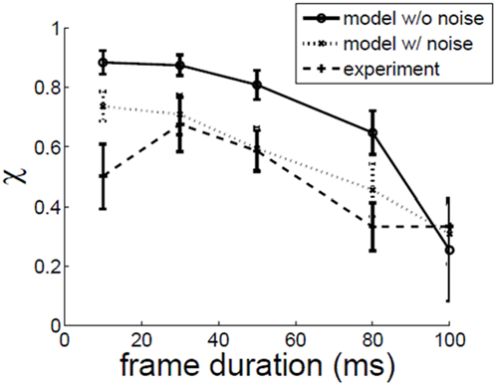
χ vs. frame duration fd. c = 0.1, ic = 7°, dd = 5 dots/degrees^2^.

Figure 6 shows that fd≈30 ms is optimal for motion perception. The same sequence of frames that evoke perception of vivid motion at fd≈30 ms, fail to evoke any perception of motion at fd ≫30 ms. The explanation proposed for the fd effect seen here is as follows. The motion computed by local motion detectors at time t is based on the spatiotemporal signal from time t-T to time t, where T≈200 ms is the temporal size of receptive fields of simple/complex cells found in the primary visual cortex [44]. When fd is too large the input is mostly constant within a window of 200 ms and so motion sensitive cells will fail to detect any motion. On the other hand, if fd is too small the input may be changing at a rate that the cells cannot handle. The bandwidth of the stimulus, viewed as a continuous-time signal, is directly proportional to the rate at which the individual racetrack frames are played. When fd is too low, the correlated dot will stay in the receptive field (RF) of a motion sensitive cell for a very brief interval of time, and will not excite the spatiotemporal RF profile of the cell.

The model results are close to that of experiment, except for the χ values at fd = 10 ms. This may be because of the high bandwidth of neurons used in the model. It is interesting to note that without noise, χ at fd = 100 ms is at the baseline zero level. If noise is added, χ rises above zero level, and matches value given by human observers. This is reminiscent of the beneficial effect noise may sometimes play in a system, by stochastically boosting a subthreshold signal in the manner of stochastic resonance [45], [46], [47].

### Effect of dot density dd

**Figure 7:**
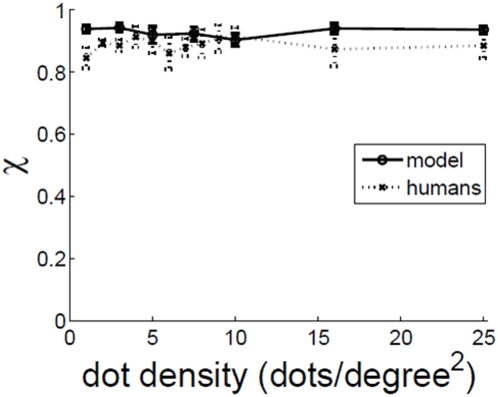
χ vs. dot density dd. c = 0.2, ic = 7°, fd = 30 ms. Model simulations done at 256×256 pixel resolution for dd>10.

Figure 7 shows the effect of varying the dot density dd in the display. Humans display a remarkable indifference to the dot density in the display. This shows that it is the relative proportion of the correlated dots that matters, not their absolute number. The experimentally observed independence of observer performance on dot density cannot be explained by models of motion perception based on matching dots or features to their nearest neighbors in the next frame [6], [7]. Such models display a marked dependence on dot density in the display according to the probability of mismatch formula [8]. As the dot density increases there are more dots per unit area, and the chances that the nearest neighbor is not the correlated partner increase.

A derivation of the probability of mismatch formula follows. A correlated dot is displaced by a distance *h* in the next frame. A nearest neighbor model operates by matching dots to their nearest neighbors in the next frame. The matching directly gives the local motion vectors, which is the output of the model. Therefore, for the correlated dot to be matched correctly to its partner, no dot should fall within a circle of radius *h* in the next frame. The probability of this happening, which is equal to the probability of no mismatch equals:
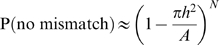
where A is the area of display, N is total number of dots in the display, N = A·dd, and we assume that dots are uniformly distributed.

Approximating 1−x as exp(−x) for x sufficiently small, and substituting A·dd for N,
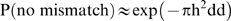
therefore,
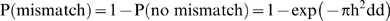
(1)which the formula given in Williams & Sekuler (1984) [8]. The probability of mismatch values for the dot density range used in Figure 7, are tabulated in Table 3. This formula makes it explicit that as the dot density increases, there would be more and more mismatches, and therefore observer performance should decrease with increase in dot density. In reality, however, observer performance is independent of dot density in the display. The Watson Ahumada motion detector is able to capture this independence as shown in Figure 7.

**Table 3:** Probability of mismatch values for dot densities in Figure 7.

Dot density (dots/degrees^2^)	Probability of mismatch = 1−exp(−πh^2^dd) (up to 4 decimal places) h = 0.37°
1	0.3495
2	0.5769
4	0.8210
8	0.9680
16	0.9990
25	1.0000

It may be noted that if some of the assumptions leading to the formula in equation (1) do not hold, the analytic form of P(mismatch) may no longer be given accurately by 1−exp(−πh^2^dd). However, the central thesis of the formula that observer performance should decrease with dot density will still remain true. This is because as the dot density increases, there are more dots per unit area, and therefore the expected separation between dots would decrease. When the expected dot separation becomes less than the hop size *h*, the matching would be dominated by mismatches, and performance would decline. It may be appropriate here to remark on the study of Grzywacz, Watamaniuk and McKee (1995) (figure 1 in their paper) [48]. It appears to me that the authors correctly simulated the Adelson Bergen model and found that it is insensitive to dot density. However, they concluded incorrectly, misguided by the probability of mismatch formula, that psychophysical results should depend on dot density.

### Effect of spatial hop size h

**Figure 8:**
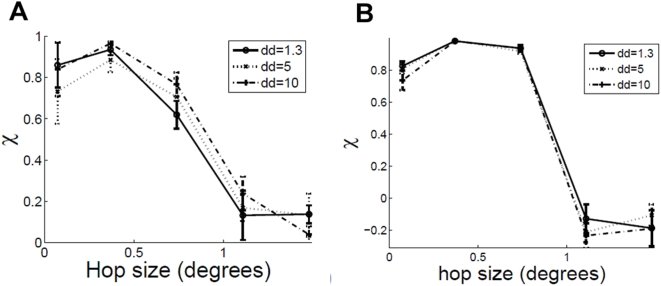
(a) χ vs. hop size h for human observers (b) χ vs. hop size for model. c = 0.4, fd = 30 ms, ic = 7°.

The hop size is the amount of displacement given to the correlated dots. By default the correlated dots are rotated by an angle of 5°. With ic = 7°, and angle subtended by outer circle fixed at 10°, this translates to average displacement of 
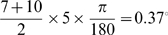
 visual angle on the eye. Figure 8  shows the effect of varying the hop size for the model and humans, at different dot densities. The correlated dots were rotated by angles of {1,5,10,15,20} degrees, corresponding to average displacements of {0.074, 0.37, 0.74, 1.11, 1.48} degrees visual angle on the eye. The curves for the model and humans are approximately similar. Note in particular that changing dot density does not produce any change in χ. The figures show that as the hop size is increased, motion disappears in the display even though the dot correlation is very high (c = 0.4). This is because if the hop size becomes greater than the RF size, motion sensitive neurons will fail to register motion. Also important is the decrease in χ if the hop size becomes too small. In this case, the spatiotemporal profile of the stimulus will not cross-correlate well with the spatiotemporal RF of motion sensitive cells.

### Effect of inner circle diameter ic

**Figure 9:**
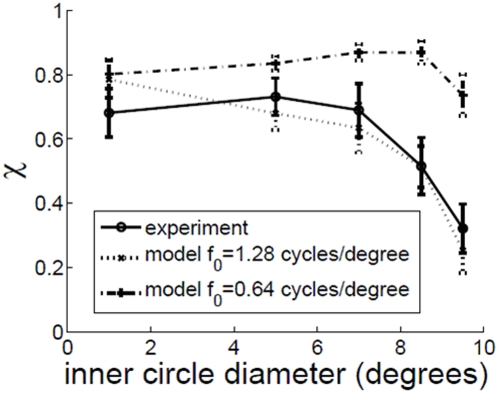
χ vs. angle subtended by inner circle diameter ic. Angle subtended by outer circle diameter is fixed at 10°. c = 0.1, dd = 5 dots/degrees^2^, fd = 30 ms. Model simulations at 256×256 pixel resolution. f_0_ denotes center frequency of Watson Ahumada sensors.

The angle subtended by the outer circle diameter is fixed at 10° in all the experiments. Figure 9 shows the effect of varying the angle subtended by the inner circle diameter ic (c = 0.1). It is seen that observer performance falls off as the angle subtended by the inner circle diameter ic is changed from 7° to 9.5°. At ic = 9.5° the annulus is very thin, and appears like a 1D ring rather than a 2D annulus. When ic is small, the noise in the display is uniformly distributed in the sense that if θ is the angle made by the noise vector, then θ is uniformly distributed from −π to +π. Denote the cross product of noise vector with the radial vector by *x* = sin(*θ*). Then 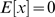, and the amount of noise is given by
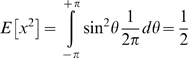



On the other hand, when ic→oc, θ is either –π/2 or +π/2 with equal probability. 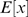 is still zero but
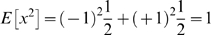
so the amount of noise has apparently doubled in this case. Model performance is seen to partially match psychophysical performance. The curve with center frequency equal to 1.28 cycles/° shows a better fit than the curve with center frequency equal to 0.64 cycles/°. Unfortunately I cannot say why the former curve shows a better fit.

### Effect of reverse contrast

**Figure 10:**
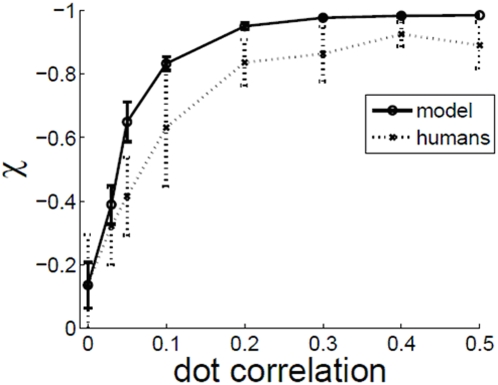
χ vs. c for contrast reversing dots. fd = 30 ms, ic = 7°, dd = 2.5 dots/degrees^2^.

If the stimulus is modified such that the correlated dots flip their polarity as they rotate, meaning black dots change to white and vice-versa, then the reverse-phi motion [49], [50], [1] takes place. It is found that the motion perceived by an observer is opposite to the physical displacement of the correlated dots. If the correlated dots move CCW(CW), observer perceives motion in CW(CCW) direction respectively. The Watson Ahumada model is able to capture this phenomenon as shown in Figure 10. If the observer perceives motion in a direction opposite to rotation of the correlated dots, the observer response is negatively correlated with the embedded motion. For this reason χ in Figure 10 is defined as the minimum value of the normalized cross correlation function between the response and input function.

**Figure 11:**
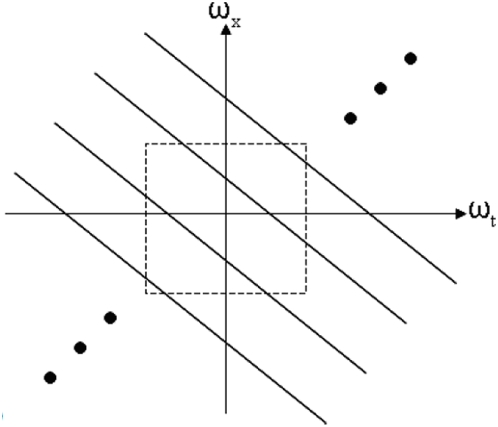
The Fourier Transform of an image undergoing coherent translational motion + periodic reverse contrast lies on infinitely many planes of the form 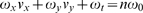, with 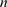 being an odd number. The dashed lines denote the window of visibility [30].

To understand why motion may be perceived in the opposite direction when dots reverse their contrast, consider the signal 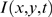. It is well known [17] that the Fourier Transform of an image undergoing coherent translational motion lies on a plane, i.e., if 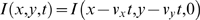 then 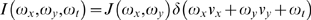 where 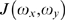 is the 2D Fourier Transform of 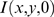, and 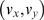 is velocity. The equation of plane is 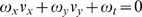. This observation yields following algorithm to determine motion in a signal 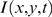: find the best fitting plane to 
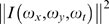
 that passes through the origin. The velocity can be read off the equation of the plane. Now consider what happens when 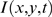 reverses its contrast every 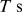. The modified signal is given by 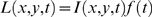, where 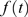 is a square wave alternating between +1 and −1 every 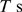. The Fourier Series of 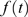 is given by 
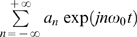
, with 
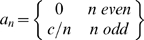
, 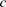 being a constant, and 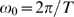. This gives 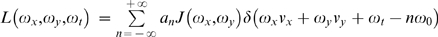. Note that 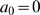. Thus, the Fourier Transform of 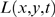 does not lie on a plane passing through the origin. Instead, the Fourier Transform of 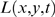 consists of infinitely many planes given by 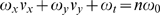 as illustrated in Figure 11. Assuming 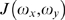 is mostly constant, the best fitting plane to 
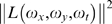
 (that also passes through the origin) is ⊥ to 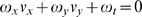. If 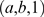 is normal of the best fitting plane, then 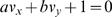. 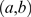 is velocity of 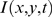 under reverse contrast. Letting 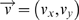, and 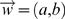 we have
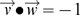



This equation can be satisfied by many 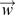. In particular 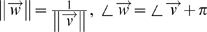 is a solution, which is motion in opposite direction to 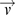. Note that 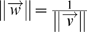 suggests that a faster moving particle should actually appear to move slower! This surprising prediction appears to be true within appropriate range. A display of alternating black and white stripes was made. The width of a stripe was 0.25°. The stripe pattern was translated to the right, and the stripes reversed their contrast after a time interval T. On viewing the display, motion was perceived in the leftward direction instead of right. With fd = T = 30 ms and a hop size of 0.125°, the pattern appeared to be moving slower than with hop size of 0.0625°. Further experiments need to be done to gather numerical data to quantify the effect.

### Model sensitivity to center position

**Figure 12:**
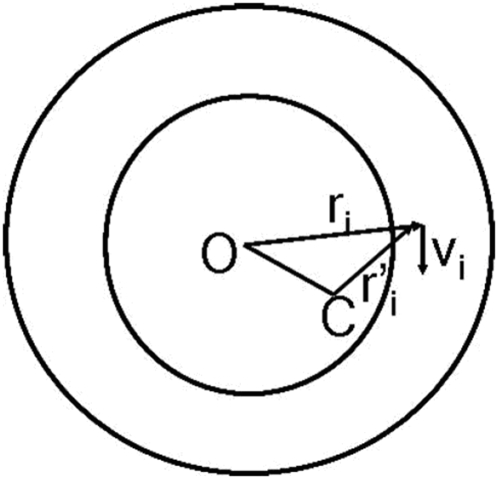
Point O represents the true center of rotation, whereas point C is the center relative to which rotary motion is computed by the model. The offset is given by 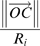 where 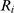 is radius of inner circle.

By definition of rotation, any measure of rotary motion has to be specified with respect to some center of rotation (more accurately the axis of rotation has to be specified). In all the results presented up till now, the center position used in the simulations was the true center of rotation of the dots. What happens if the true center of rotation is not accurately known, as must be the case in reality? Figure 12 shows a schematic in which point O is the true center relative to which the correlated dots are rotating, and point C is the center relative to which rotary motion is computed by the model. 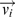 is a motion cue. The rotary motion relative to the true center O is given by 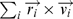, whereas the rotary motion relative to C is given by 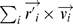. We have
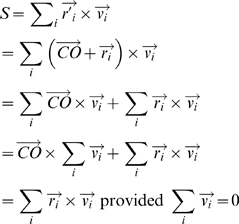



**Figure 13:**
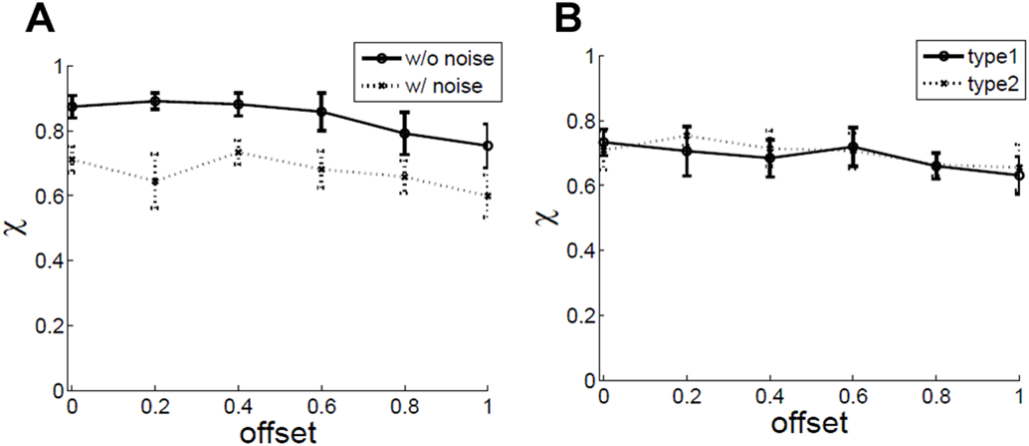
χ vs. center relative to which rotary motion is computed (a) full 360° annulus is visible (b) only 90° of annulus is visible. Type1 – a single 90° sector is visible. Type 2 - two sectors located diametrically opposite to each other, and each 45° in size, are visible. Both curves are for the model.

The condition 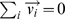 is true in case of the racetrack. The uncorrelated dots are uniformly distributed and generate motion cues in all directions with equal probability. The correlated dots generate motion cues in tangential direction, which when summed over the entire 360° annulus add up to zero. The expected value of 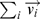 is thus zero. Therefore it seems accurate knowledge of position of the true center relative to which rotation occurs is not needed. Figure 13(a) shows model sensitivity to knowledge of true center position. The rotary motion is computed by the model relative to a point C that is offset from the true center O. The offset is given by 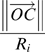 where R_i_ is radius of inner circle. Two curves are shown: in one there is no noise added to the model, i.e., n(t) = 0, and in the other GWN equal to the default value of σ(GWN)/σ_0_ = 6 is added to the model. It can be seen that the χ values are not affected much by uncertainty in knowledge of true center position, and start to deteriorate only when the offset becomes very large. This may explain the experimentally observed position invariance of MST(d) cells, the fact that the cells are insensitive to where in their RF rotation occurs [9].

It seems that when only a sector of the racetrack is made visible, the condition 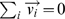 may not hold true because of the correlated dots. However, if two sectors located diametrically opposite to each other are displayed then 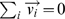. Figure 13(b) shows χ vs. offset for the two cases: type1 when only a single 90° sector is made visible, and type2 when two sectors located diametrically opposite to each other, and each 45° in size, are displayed. Interestingly the model is still robust enough to the offset even when only a sector of the racetrack is displayed, irrespective of whether it is type1 or type2.

### Effect of displaying only a sector

**Figure 14:**
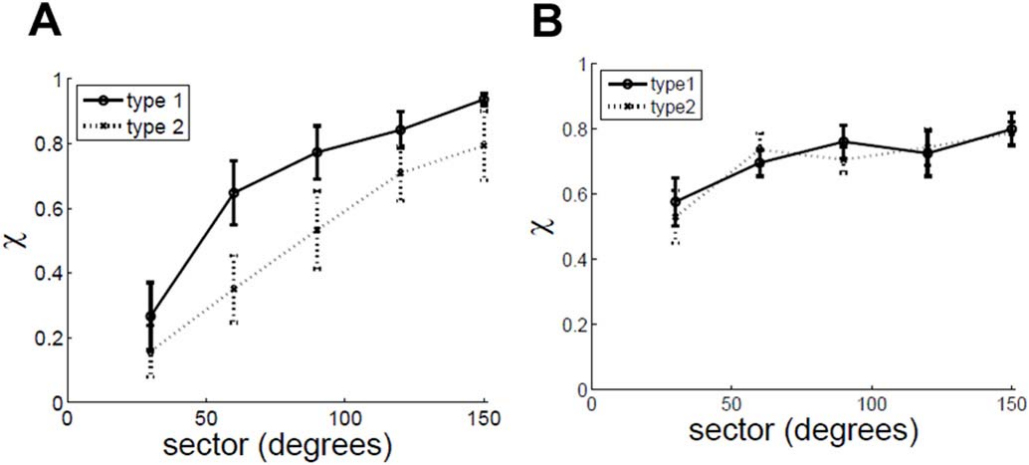
χ vs. sector. In case of type 1 only one sector is displayed, whereas in case of type 2 two sectors located diametrically opposite to each other, and each half the size of sector in type 1, are displayed. (a) human performance (b) model performance.

Figure 14(a) shows the effect of displaying only a sector of the complete annulus on human observers. Two cases are considered. In type1, a single sector is shown that is randomly positioned. In type2, two sectors located diametrically opposite to each other, and each half the size of the sector in type1, are displayed. It is seen that χ increases monotonically as the sector size increases. It is interesting to note that there is a significant difference in χ for the two cases, even though the total area displayed is the same in the two cases. The corresponding data for the model is shown in Figure 14(b). The model shows an increase in χ with sector size. However, there is no difference between type1 and type2 for the model.

### Effect of inserting random frames

**Figure 15:**
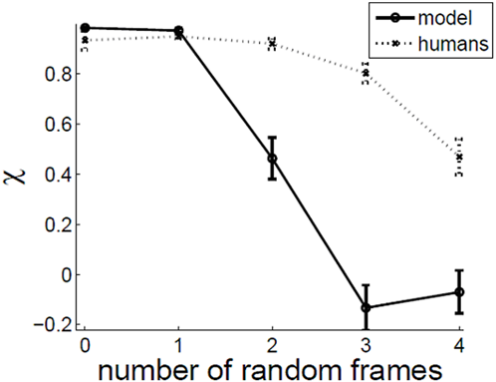
Effect of inserting K random frames between correlated frames. c = 0.5, fd = 30 ms, dd = 5 dots/degrees^2^, ic = 7°.

Figure 15 shows effect of inserting K random frames between every pair of correlated frames in the stimulus. It is seen that observer performance does not fall to zero abruptly, but decreases in a graceful manner showing that the human visual system takes multiples frames into consideration when estimating motion. The model performance also does not fall to zero abruptly, but degrades much more rapidly than human performance.

### Dipoles

**Figure 16:**
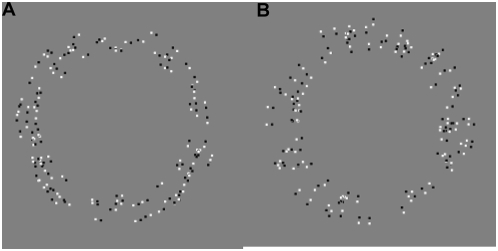
(a) tangential dipoles (b) radial dipoles. Center-to-center spacing  = 17′ in both cases.

Instead of displaying dots in an annulus, each dot can be split into two dots – one black and one white forming a dipole. This results in what has been termed as the anti-Glass pattern [51]. The *c = 0* case creates a powerful motion illusion that has been previously investigated [35]. The addition of dipoles introduces several new parameters:

1. the dipole spacing s,
2. the black to white intensity ratio bwir defined as 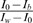 where I_0_, I_b_, I_w_ are luminance of background (fixed at 10.8 cd/m^2^), black and white dots respectively,
3. the dipole orientation: tangential or radial as in Figure 16

**Figure 17:**
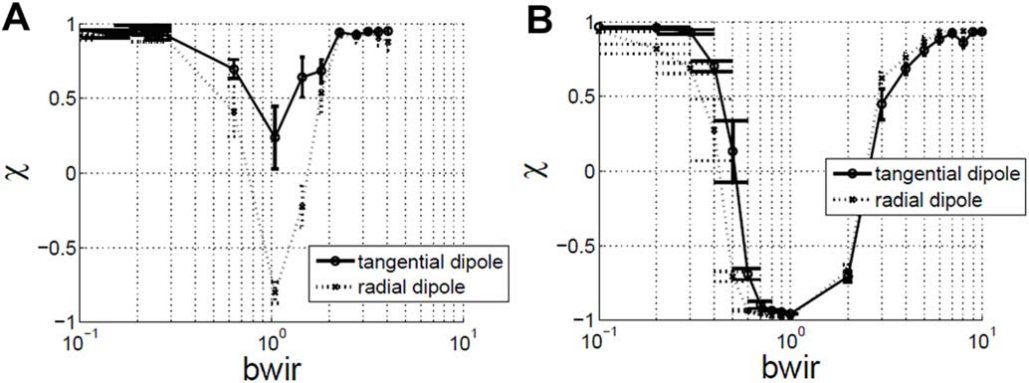
χ vs. bwir (black to white intensity ratio). Dipole spacing  = 6′, c = 0.5, dd = 2.5 dots/degrees^2^, RC ON, (a) human observers (b) Watson Ahumada model with simulations at 256×256 pixel resolution.

Complex patterns of motion are perceived with dipoles in the display, e.g., if dipoles are oriented radially there is tendency to observe radial pulsating motion, even if dipoles are actually rotating with significant rotary motion. Depending upon the parameter settings, motion in opposite directions is also seen. It can become difficult to assign a single motion direction to the whole display, although there is no doubt that there is motion in it. Let RC (reverse contrast) ON denote the setting that if a dipole is correlated, then black changes to white and vice-versa in the next frame. With RC ON, the perception of motion can switch from normal phi to reverse phi depending upon dipole spacing. This section reports results of an experiment investigating χ vs. bwir with center-to-center spacing equal to six minutes, *c = 0*, and RC ON. The results are summarised in Figure 17. As can be seen the model is able to capture some aspects of psychophysical behavior, but not all of it. In Figure 17, the definition of χ is modified as follows. Let χ_+_ denote maximum value of normalized cross correlation function, and χ_−_ denote minimum value of normalized cross correlation function. If |χ_+_|>|χ_−_|, χ = χ_+_, otherwise χ = χ_−_. When |χ_+_|>|χ_−_|, observer perceives motion in the direction of displacement of correlated dots and therefore χ is defined to be equal to χ_+_ in this case. By similar reasoning, χ is defined to be χ_−_ for the other case.

## Discussion

Although this paper shows that the Watson Ahumada motion detector does a good job at detecting motion in random dot kinematograms (RDKs) consonant with human psychophysical performance, it remains to be seen how well it would perform on real world imagery. The challenge here is that although it is straightforward to run the model on real world test cases, how do we accurately measure the optical flow perceived by humans on these test cases? Computer vision papers characterise optical flow performance of a model by either using synthetic image sequences designed to mimic the real world, or using real world image sequences in which the motion of the camera is carefully calibrated [52]. However, as we have already seen in this paper: 1. the same sequence of image frames can produce different perception depending on frame rate, 2. the human visual system takes multiple frames into consideration when determining motion. In the light of these remarks, it is not immediately obvious what the ground truth optical flow (ground truth being defined as the flow perceived by a human) would be for the test cases mentioned above. These caveats should be borne in mind while attempting a performance characterisation of the Watson Ahumada model using the computer vision paradigm. I have placed some preliminary work running the Watson Ahumada model on real world imagery online as a proof of concept [41].

The neurophysiological plausibility of a model is likely to attract heavy debate. Krekelberg (2008) has provided a comprehensive discussion on the biological plausibility of the Reichardt, Adelson Bergen, and gradient based motion detectors [13]. With respect to the Watson Ahumada model, DeAngelis et. al. (1995) and others have found that the Watson Ahumada filters provide an accurate model of simple cell receptive fields (RFs) [44]. Quoting DeAngelis et. al. [53]:

“Rather, simple cell RFs in the joint space-time domain appear to be fit well by a model first proposed by Watson and Ahumada … Based on the Watson-Ahumada formulation, we have modelled space-time RFs of simple cells, as the weighted sum of two space-time separable subunits in a quadrature relationship. This model formulation provides a remarkably good fit to the data from most cells, regardless of their degree of space-time inseparability … In conclusion, to account for space-time RFs of simple cells that differ widely in the degree of space-time inseparability, at least two separable subunits appear necessary as modelled by Watson and Ahumada.”

Although there are similarities between the Watson Ahumada motion detector and the Adelson Bergen motion detector, which is usually the *de facto* motion detection mechanism used in studies of visual motion perception, there are also some differences. The Adelson Bergen motion detector measures how much power the stimulus has within a spatiotemporal frequency band. Thus a detector tuned to (ω_x0_,ω_y0_,ω_t0_) effectively samples the power spectrum of the stimulus within the vicinity of (ω_x0_,ω_y0_,ω_t0_). Such detectors have been proposed as models of V1 complex cells [1], [21]. The responses of multiple such detectors tuned to different spatiotemporal frequencies are pooled to determine the best fitting plane in the frequency domain [18], [19]. The best fitting plane defines the motion of the stimulus [17]. This processing, although still debatable, is believed to occur in MT. In contrast, with respect to the Watson Ahumada motion detector, information about the motion of the stimulus is encoded in the (most dominant) temporal frequency of oscillation of detector response as per the equation:
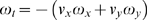



The temporal frequencies of oscillation of different detectors tuned to different (ω_x_,ω_y_) are measured, and then above relationship is used to determine the motion of the stimulus. The neural locus of the stages that perform this computation is unclear. Also unclear is the relationship of the model to what we do know about motion processing in the brain beyond the first stage of spatiotemporal filtering. For example, the model does not state how simple V1 neuron outputs could be combined to generate speed tuned V1 complex and MT cells [54], [55]. Perrone (2005) has put forward a model that explains how the magnitude of the Fourier transform of simple V1 neuron responses can be combined to generate the magnitude of the Fourier transform of a speed tuned neuron [56]. The input V1 neurons that Perrone's model uses are based on the Watson Ahumada filters.

It may be worthwhile to mention that the Watson Ahumada model has been proposed as a model of primary motion sensing mechanisms, what Cavanagh (1991) called passive motion detectors in his paper [57]. The human visual system is a complex parallel distributed system in which modules interact with each other and do not function in isolation, e.g., it is widely accepted now that motion perception interacts with form perception, a view that was not always held in this field. The interactions between modules can give rise to phenomenon that cannot be explained by either module alone. Benton, O'Brien, & Curran (2007) have recently provided example of a fractal rotation stimulus in which rotation is perceived within any arbitrary window applied to the stimulus [58]. The authors assert that the fact that observers can readily perceive fractal rotation is a clear example of a stimulus in which motion extraction is dependent upon the prior analysis of some spatial property (which happens to be the orientation in case of fractal rotation). The omega effect itself is believed to occur because of interactions between form and motion processing circuits in the brain. Although there are growing examples of such stimuli that point to interactions between form and motion, little is known about how these interactions occur. To my knowledge no quantitative model has been put forward to explain these interactions.

In conclusion, the contribution of this paper is to present a performance characterisation of the Watson Ahumada model of human visual motion sensing. The model performance is seen to match human performance with respect to most parameters. It is able to explain some key and important parts of the psychophysical data such as independence of observer performance to dot density in the display, and decrease of observer performance with frame duration of the display. The model insensitivity to the center position relative to which rotary motion is computed, together with the vector analysis presented in the paper, may explain the experimentally observed position invariance of MST(d) cells. In addition, this paper shows that the omega effect of Rose & Blake (1998) is a truly bistable illusion. Although the display of random dots triggers perception of rotary motion, the direction of motion perceived is independent of what dot pattern is shown. The time interval between spontaneous reversals in perceived direction is lognormally distributed as is the case for most bistable illusions. Therefore the processes that give rise to this illusion may be the same processes that underlie much of other bistable phenomenon.

## Supporting Information

Movie S1 A movie of the racetrack for c = 0.
(8.63 MB AVI)

Movie S2 A movie of the racetrack for c = 0.3.
(8.02 MB AVI)

Movie S3 A movie of the racetrack for c = 0.5.
(8.94 MB AVI)
